# BES1 regulates the localization of the brassinosteroid receptor BRL3 within the provascular tissue of the Arabidopsis primary root

**DOI:** 10.1093/jxb/erw258

**Published:** 2016-08-10

**Authors:** Jorge E. Salazar-Henao, Reinhard Lehner, Isabel Betegón-Putze, Josep Vilarrasa-Blasi, Ana I. Caño-Delgado

**Affiliations:** Department of Molecular Genetics, Centre for Research in Agricultural Genomics (CRAG), CSIC-IRTA-UAB-UB, Barcelona E-08193, Spain

**Keywords:** BES, *BRL3*, brassinosteroid, quiescent center, root, vascular.

## Abstract

Brassinosteroid (BR) hormones are important regulators of plant growth and development. Recent studies revealed the cell-specific role of BRs in vascular and stem cell development by the action of cell-specific BR receptor complexes and downstream signaling components in *Arabidopsis thaliana*. Despite the importance of spatiotemporal regulation of hormone signaling in the control of plant vascular development, the mechanisms that confer cellular specificity to BR receptors within the vascular cells are not yet understood. The present work shows that BRI1-like receptor genes 1 and 3 (*BRL1* and *BRL3*) are differently regulated by BRs. By using promoter deletion constructs of *BRL1* and *BRL3* fused to GFP/GUS (green fluorescent protein/β-glucuronidase) reporters in Arabidopsis, analysis of their cell-specific expression and regulation by BRs in the root apex has been carried out. We found that *BRL3* expression is finely modulated by BRs in different root cell types, whereas the location of *BRL1* appears to be independent of this hormone. Physiological and genetic analysis show a BR-dependent expression of *BRL3* in the root meristem. In particular, *BRL3* expression requires active BES1, a central transcriptional effector within the BRI1 pathway. ChIP analysis showed that BES1 directly binds to the BRRE present in the *BRL3* promoter region, modulating its transcription in different subsets of cells of the root apex. Overall our study reveals the existence of a cell-specific negative feedback loop from BRI1-mediated BES1 transcription factor to *BRL3* in phloem cells, while contributing to a general understanding of the spatial control of steroid signaling in plant development.

## Highlight

Brassinosteroid-regulated transcription factor BES1 targets the *BRL3* receptor gene and finely modulates its transcription in the vascular and stem cells of the Arabidopsis primary root.

## Introduction

Brassinosteroids (BRs) are polyhydroxylated plant steroid hormones that were first identified by their ability to elongate plant stems when applied exogenously (Grove *et al.*, 1979; Mandava, 1988). The identification of mutants affected in BR synthesis or signal transduction has revealed an essential role for this hormone in cell elongation and differentiation (Fukuda, 1997; Yamamoto *et al.*, 1997). So far, BRs have been reported to be involved in the regulation of multiple developmental and physiological processes such as cell division, elongation, and also the differentiation of vascular and stem cells, among others (Fabregas and Caño-Delgado, 2014). The BRASSINOSTEROID INSENSITIVE 1 (*BRI1*) gene was identified by a genetic screening of a BR-insensitive loss-of-function mutant performed in the model plant Arabidopsis (*Arabidopsis thaliana*) (Clouse, 1996). This mutant exhibited severe dwarfism with characteristic dark green and epinastic leaves and a reduced apical dominance and fertility (Szekeres *et al.*, 1996; Clouse, 1996; Kauschmann *et al.*, 1996; Li *et al.*, 1996; Azpiroz *et al.*, 1998; Choe *et al.*, 1999, 2001; Noguchi *et al.*, 1999). The *BRI1* gene encodes a membrane-localized leucine-rich repeat receptor-like kinase (LRR-RLK) comprising an extracellular LRR domain, a single transmembrane domain, a juxtamembrane domain, a cytoplasmic serine/threonine kinase domain, and a C-terminal regulatory region (Li and Chory, 1997). Genetic and biochemical assays have demonstrated that the BRI1 receptor complex directly bind BRs with high affinity (Wang *et al.*, 2001). This occurs via direct binding of BRs to the extracellular domain of the LRR-RLK proteins at the cell membrane (Wang *et al.*, 2001; Kinoshita *et al.*, 2005).

Recent structural studies have confirmed that brassinolide (BL) binds to the BRI1 plasma membrane receptor through a 70 amino acid island domain located within the extracellular domain of BRI1, creating a surface pocket for ligand binding (Hothorn *et al.*, 2011; She *et al.*, 2011). Upon direct binding of BL to this extracellular domain (Kinoshita *et al.*, 2005), BRI1 forms a heterodimer with its co-receptor BAK1 ASSOCIATED RECEPTOR KINASE 1/SOMATIC EMBRYOGENESIS RECEPTOR KINASE 3 (BAK1/SERK3), yet another LRR-RLK (Li *et al.*, 2002; Nam and Li, 2002; Russinova *et al.*, 2004). Subsequently, the BR signal is modulated intracellularly in a phosphorylation- and dephosphorylation-dependent manner, ending in the de-phosphorylation of the BRI1-EMS-SUPPRESSOR 1 (*BES1*) and BRASSINAZOLE RESISTANT 1 (*BZR1*) genes (Li *et al.*, 2002; Wang *et al.*, 2002; Yin *et al.*, 2002; Mora-García *et al.*, 2004; Gampala *et al.*, 2007 ). Both BES1 and BZR1 are members of a plant-specific family of basic-helix–loop–helix (bHLH) transcription factors that act as homo- or heterodimers (Yin *et al.*, 2002; He *et al.*, 2005; Yu *et al.*, 2008; Li *et al.*, 2009). The gain-of-function mutant *bes1-D* is known to be constitutively active mutant independent of BR and BRI1 signaling and able to suppress *bri1* phenotypes. The accumulation of de-phosphorylated BES1 protein in the nucleus, where BES1 is activating its target genes, is higher in *bes1-D* lines than in the wild type (Wang *et al.*, 2002; Yin *et al.*, 2002).

Detailed analysis of promoter elements indicated that both BES1 and BZR1 are able to bind BR response elements (BRREs) as well as E-boxes (Sun *et al.*, 2010; Yu *et al.*, 2011). Binding of BES1 to BRREs was shown to be much stronger than to E-boxes, since efficient BES1 binding to the latter needs a partner (Yin *et al.*, 2005). While BRREs are mostly enriched in BR-repressed genes, E-box elements are mostly enriched in BR-induced genes (Sun *et al.*, 2010; Yu *et al.*, 2011). Additionally, it was previously suggested that BZR1 acts as a transcriptional repressor (He *et al.*, 2005) while BES1 acts as a transcriptional activator (Yin *et al.*, 2005). However, recent genome-wide ChIP analysis showed that both BZR1 and BES1 function either as activators or repressors. BES1 and BZR1 regulation of downstream targets is most probably determined by additional promoter sequence elements and/or BES1- and BZR1-interacting proteins (Sun *et al.*, 2010; Ye *et al.*, 2011; Yu *et al.*, 2011).

In addition to BRI1, three additional LRR-RLK proteins have been identified as BRI1 homologs named BRI1-LIKE RECEPTORS 1, 2, and 3 (BRL1, BRL2 and BRL3) (Caño-Delgado *et al.*, 2004). Unlike *BRL2*, previously described as VASCULAR HIGHWAY 1 (VH1) (Clay and Nelson, 2002), *BRL1* and *BRL3* encode membrane-localized receptors able to bind BL with high affinity. The expression of the *BRL1* and *BRL3* genes under the *BRI1* promoter reverts the phenotypic defects in the *bri1* mutant, demonstrating that both *BRL1* and *BRL3* are functional BR receptor genes (Caño-Delgado *et al.*, 2004; Zhou *et al.*, 2004). In contrast to *BRI1* that is widely expressed in plants, *BRL1* and *BRL3* exhibit an enriched expression in the vasculature (Caño-Delgado *et al.*, 2004). Biochemical purification of the BRL3 complex has addressed the cellular specificity of BR receptor complexes in plants (Fàbregas *et al.*, 2013), thus suggesting that the localization of these receptors accounts for specific cellular functions (Fàbregas *et al.*, 2013). It has been proposed that the BRI1, the BRL1, and the BRL3 receptors signal together in BR-mediated root growth and quiescent center (QC) division dynamics, although whether this interaction occurs at the receptor level or by a downstream signaling component is not yet established.

In this study, the 5' intergenic region of *BRL1* (*ProBRL1*) and *BRL3* (*ProBRL3*) has been analyzed, to identify *cis*-acting elements required for QC and vascular-specific expression patterns. The *ProBRL1* expression seemed to be BR independent, whereas the expression of *ProBRL3* showed a BR dose-dependent spatial expression pattern. This analysis reveals that binding of the BR-activated transcription factor BES1 to a *cis*-acting BRRE located at base pair −1441 of the *BRL3* promoter controls the spatial localization of the receptor in plants. Overall this study advances the idea that BRI1 and BRL3 receptor signals are interconnected by BES1, which provide the cellular specificity for *BRL3* transcription in specific cells.

## Materials and methods

### Plant material and growth conditions

*Arabidopsis thaliana* Columbia ecotype (Col-0) was used to generate all the *ProBRL3::GUS* and *ProBRL1::GUS* transgenic plants.The *bes1-D* mutant introgressed into the Col-0 ecotype was used in this study (Ibanes *et al.*, 2009). Seeds were surface sterilized in 35% sodium hypochlorite, vernalized for 72h at 4 °C in the dark, and grown on plates containing 1× Murashige and Skoog (MS) salt mixture, 1% sucrose, and 0.8% agar in the absence or presence of different concentrations of BL (C28H48O6; Wako, Osaka, Japan). In the case of the 35S::*bes1-D*:GR transgenic plants, the agar was supplemented with 1 µM dexamethasone. Plants were grown under fluorescent light (12h light/12h dark cycles) for 6 d prior to analysis.

### *In silico* analysis of the promoters

The search for pre-determined regulatory promoter elements was done using the program DNA-pattern (Thomas-Chollier *et al.*, 2008).

### Generation of promoter constructs

To generate the various *BRL3* and *BRL1* promoter fusion constructs, Invitrogen’s Gateway technology was used. In the first reaction step, pDONR221 or pDONR207 was used to generate entry clones. In the second step, the destination vectors pHGWFS7 [(green fluorescent protein/β-glucuronidase) GFP/GUS] and pGWB635 (firefly luciferase) (Nakamura *et al.*, 2010) were used to generate the expression clones. Transgenic plants (GFP/GUS) from 10 independent T_4_ homozygous lines were selected by hygromycin resistance and homozygous plants were used for expression pattern analysis. In addition, the constructs 35S::*bes1-D*:GFP (Vilarrasa-Blasi *et al.*, 2014) and 35S::*bes1-D*:GR were generated by using the destination vector pB7m34GW (Karimi *et al.*, 2007). Primers used in the cloning of the above constructs are listed in Supplementary Table S1 at *JXB* online).

### Histology and microscopy

For GUS detection, 6-day-old seedlings were immersed in ice-cold 90% (v/v) acetone, incubated for 20min on ice, rinsed twice in dH_2_O, infiltrated with GUS [100mM sodium phosphate buffer (pH 7.2), 10mM sodium EDTA, 0.1% Triton X-100, 1mg ml^–1^ 5-bromo-4-chloro-3-indolyl-β-d-glucuronide (Xgluc; Duchefa, Haarlem, The Netherlands), 10mM potassium ferrocyanide and potassium ferricyanide] and incubated at 37 °C for 15h in the dark. Samples were rinsed three times in dH_2_O and treated with 70% ethanol. Stained roots were visualized with an AxioPhot (Zeiss, Jena, Germany) microscope. For a cell type-specific expression analysis of *ProBRL1::GUS* and *ProBRL3::GUS* within the root meristem, GUS-stained seedlings were subsequently immersed in 10% acetic acid supplemented with 50% MetOH solution and stained using a modified Pseudo-Schiff (mPS)-propidium iodide (PI) staining method (adapted from Truernit *et al.*, 2008).

To analyze the GFP localization in *ProBRL3::GFP* lines, 6-day-old roots were stained in 10 μg ml^–1^ PI and visualized after excitation by a Kr/Ar 488nm laser line. PI and GFP were detected with a 570–670nm and a 500–545nm band-pass ﬁlter, respectively. An FV 1000 confocal microscope (Olympus, Tokyo, Japan) was used throughout the study.

### Luciferase expression assays

Arabidopsis protoplasts were isolated as previously described (Sheen, 2002) and transfected with different *ProBRL3::LUC* promoter fusions (pGWB635 vector), 35S::*bes1-D*:GFP or 35S::GFP and 35S::Renilla (PHTT672, from Pioneer) (Morohashi *et al.*, 2012). For the expression assay *per se*, the Dual-Luciferase Reporter Assay System (Promega, Madison, WI, USA) was used. The bioluminescent signal was measured using a luminometer Centre LB 960 (Berthold). The data were normalized for Renilla activity. After normalization, the fold change was calculated as the ratio between each particular treatment and the treatment with the promoter constructs without transcription factor (Schagat *et al.*, 2007). For each experiment, three technical and three biological replicates were used.

### ChIP assays

35S::*bes1-D*:GFP (Vilarrasa-Blasi *et al.*, 2014) and Col-0 plants were grown in 1/2 MS (12h light/12h dark cycles) for 6 d. Seedlings were fixed with 1% formaldehyde and nuclei were extracted according to Deal and Henikoff (2011). ChIP experiments using anti-GFP antibodies were performed according to Gendrel *et al.* (2005). Detection of PCR products was performed using Absolute qPCR SYBR Green mix (Thermo Scientific) in a Biorad thermocycler. Two different biological replicas were performed for each region of interest. The ChIP-quantitative PCR (qPCR) data were analyzed using the Percent Input Method (Nagaki *et al.*, 2003). With this method, signals obtained from the ChIP are divided by signals from an input sample. This input sample represents the amount of chromatin used in the ChIP. Primers used for qPCR are listed in Supplementary Table S1.

## Results

### Promoter deletion analysis of *BRL3* and *BRL1* receptor genes

**Fig. 1:**
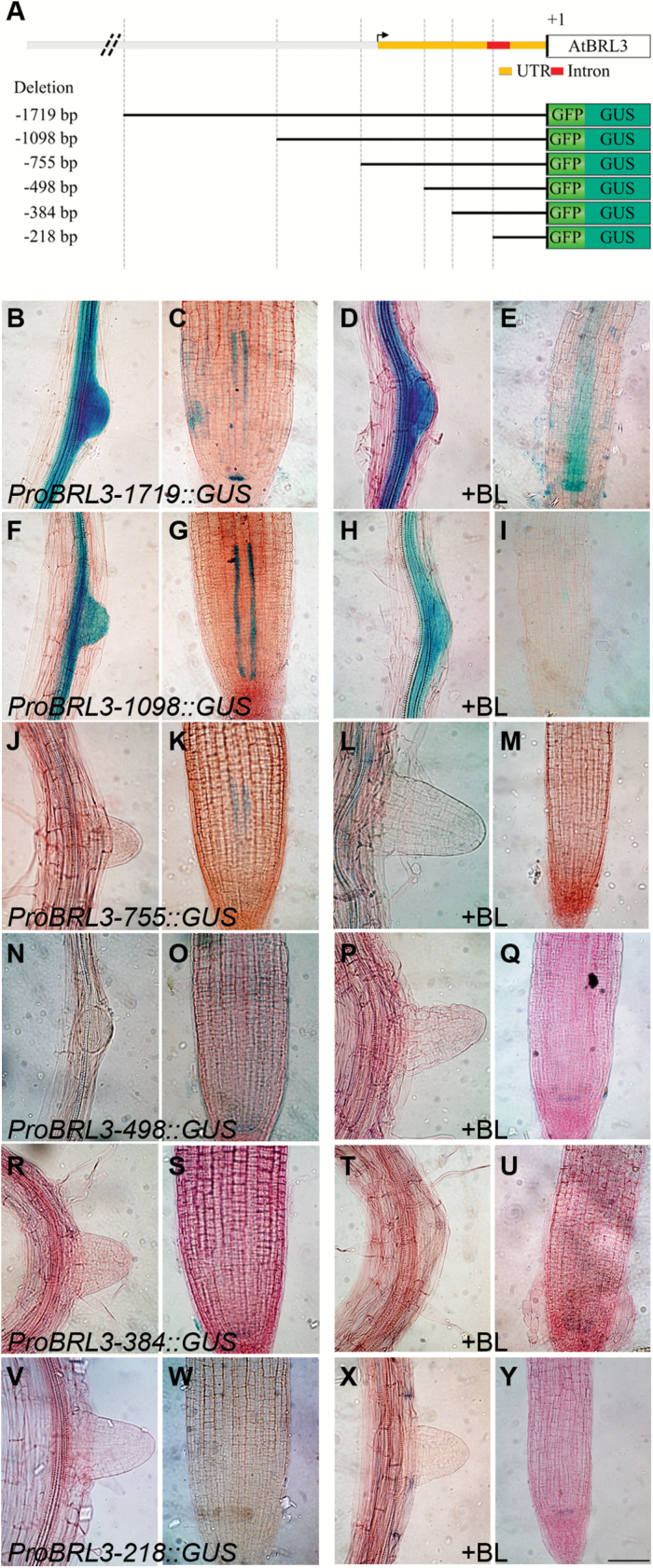
Promoter deletion analysis of *ProBRL3* in the root. (A) Schematic diagram of the 5'-flanking regions of *ProBRL3*. The figure represents part of the 5'-flanking region of *ProBRL3* including the 5'-UTR and an intron, labeled in orange and red, respectively. The lower part represents the deletion constructs generated fused to the reporter genes GFP and GUS. (B–Y) Histochemical GUS assay in the root differentiation and meristematic zone of 6-day-old *ProBRL3* transgenics with and without 4nM BL treatment for 48h. Scale bar=125 µm. (B, C) *ProBRL3-1719::GUS* showed expression in regions where lateral roots emerge, in the differentiation zone, in the two protophloem cell files, and in the QC within the meristem. (F, G) *ProBRL3-1098::GUS* showed expression in regions where lateral roots emerge, in the differentiation zone, and in the two protophloem cell files. (J, K) *ProBRL3-755::GUS* showed quite similar expression to *ProBRL3-1098::GUS* although the expression was weaker, especially in the two protophloem cell files and in the differentiation zone. (N, O) *ProBRL3-498::GUS*, (R, S) *ProBRL3-384::GUS*, and (V, W) *ProBRL3-218::GUS* showed no expression in the root. After treatment with BL (D, E) *ProBRL3-1719::GUS* showed expression in regions where lateral roots emerge, in the differentiation zone and in the QC. (E) Expression of the two protophloem cell layers is expanded and showed expression in the stele. (H, I) *ProBRL3-1098::GUS* showed expression in regions where lateral roots emerge and in the differentiation zone, and (I) a significantly repressed expression pattern in the two protophloem cell files. (L, M) *ProBRL3-755::GUS* showed quite similar expression to *ProBRL3-1098::GUS* and also exhibited (M) significantly reduced expression in the two protophloem cell files. (P, Q) *ProBRL3-498::GUS* and (T, U) *ProBRL3-384::GUS* did not show any difference from the untreated lines analyzed. (X, Y) *ProBRL3-218::GUS* was not expressed in any tissue analyzed.

Previous GUS reporter gene assays in transgenic Arabidopsis lines using a 750bp *BRL3* promoter fragment and a 1.72kb *BRL1* promoter fragment, respectively, showed an overlapping expression pattern for both genes in the plant vascular tissue (Caño-Delgado *et al.*, 2004). To identify *cis*-elements important in the regulation of *BRL3* and *BRL1*, promoter::GUS truncations were generated and used to analyze their expression (Fig. 1A; Supplementary Fig. S1A).

The *ProBRL3::GUS* expression in the root was analyzed in 6-day-old seedlings of two representative independent T_4_ homozygous line*s* generated for each construct. The *ProBRL3-1719::GUS* construct showed GUS expression in the root differentiation zone, in lateral root primordia, in the two protophloem cell files, and in the QC within the root meristematic zone (Fig. 1B, C). Removal of a 621bp region (*ProBRL3-1098::GUS*) eliminated expression in the QC, while expression in the root differentiation zone, in lateral root primordia, and in the two protophloem cell files was still visible (Fig. 1F, G). In the next shorter promoter deletion construct, *ProBRL3-755::GUS*, reduced expression was detected in the two protophloem cell files and in the differentiation zone, whereas in lateral root primordia GUS staining was lost (Fig. 1J, K). Subsequent analysis of the *ProBRL3-498::GUS*, *ProBRL3-384::GUS*, and *ProBRL3-218::GUS* lines revealed that *BRL3* expression in the root was completely lost (Fig. 1N, O, R, S, V, W). These results indicate that the *BRL3* promoter requires a minimal promoter length of 755bp for proper *BRL3* expression in the root vascular tissue, although the expression pattern differs slightly from the one detected in *ProBRL3-1719::GUS* transgenic lines. The loss of *BRL3* expression in emerging lateral roots in *ProBRL3-755::GUS* transgenics suggests that additional relevant elements may exist within the region betrween base pairs 1098 and 755. Finally, elements within the 5'-flanking region between base pairs −1719 and −1098 are controlling *BRL3* expression in the QC.

**Fig. 2:**
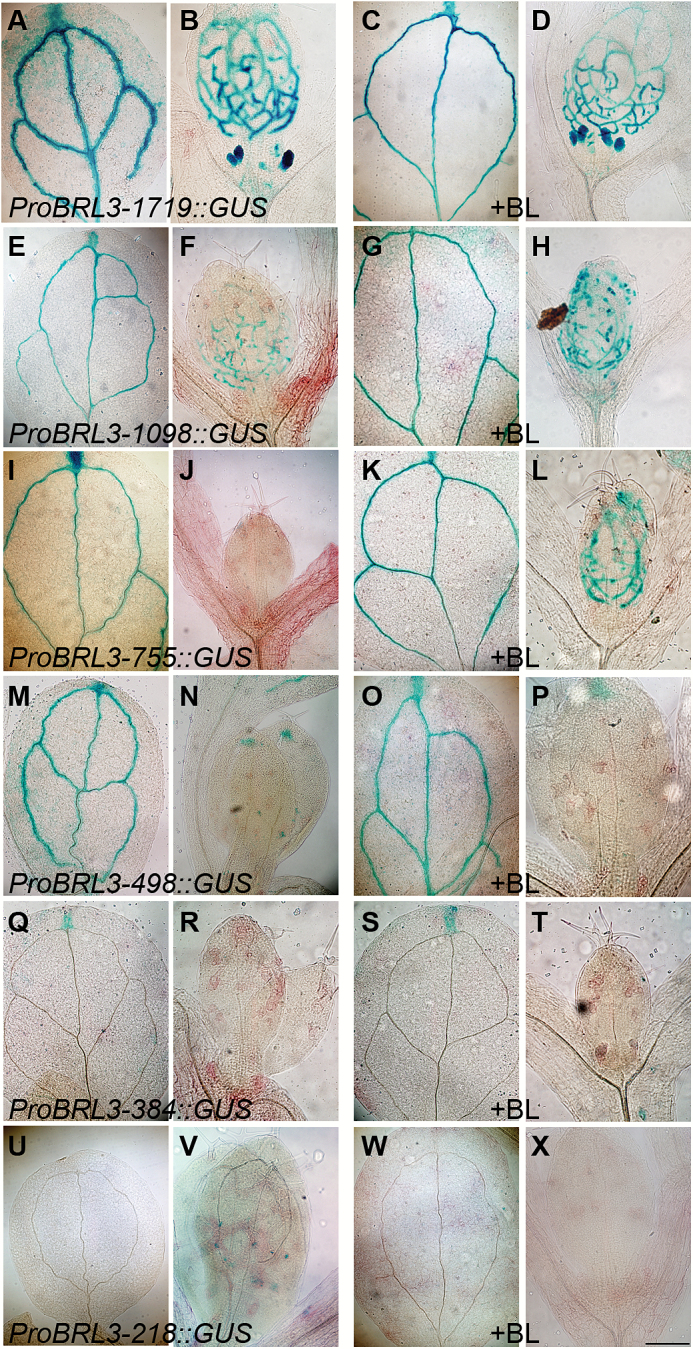
Promoter deletion analysis of *BRL3*. (A–X) Histochemical GUS assay in cotyledons and the shoot apical meristem (SAM) of 6-day-old *ProBRL3* transgenics with and without 4nM BL treatment for 48h. Scale bar=125µm. (A, B) *ProBRL3-1719::GUS* showed expression in the veins and the tip of the cotyledons and in the SAM. (E, F) *ProBRL3-1098::GUS*, (I, J) *ProBRL3-755::GUS*, and (M, N) *ProBRL3-498::GUS* showed expression in the veins and the tip of the cotyledons. (Q, R) *ProBRL3-384::GUS* was expressed only in the tip of the cotyledon. (U, V) *ProBRL3-218::GUS* did not show any expression in the tissues analyzed. After treatment with BL, an alteration in the expression pattern of *BRL3* in the cotyledons and in the SAM was not observed. (C, D) *ProBRL3-1719::GUS* showed expression in the veins and the tip of the cotyledons and in the SAM. (G, H) *ProBRL3-1098::GUS*, (K, L) *ProBRL3-755::GUS*, and (O, P) *ProBRL3-498::GUS* showed expression in the veins and the tip of the cotyledons. (S, T) *ProBRL3-384::GUS* showed only expression in the tip of the cotyledons. (W, X) *ProBRL3-218::GUS* was not expressed in any tissue analyzed.

In addition, *ProBRL3-1719::GUS* constructs displayed *BRL3* expression in the vascular tissue, the tip of the cotyledons, and in the shoot apex (Fig. 2A, B). In *ProBRL3-1098::GUS*,*ProBRL3-755::GUS*, and *ProBRL3-498::GUS* transgenic lines, *BRL3* expression was lost in the shoot apex, but still visible in the vascular tissue and also in the tip of the cotyledons (Fig. 2E, F, I, J, M, N). The *ProBRL3-384::GUS* plants only showed expression at the tip of the cotyledon (Fig.2Q, R). However, the shortest construct generated, *ProBRL3-218::GUS*, shows a complete loss of the GUS reporter activity (Fig.2U, V). Thus, *ProBRL3::GUS* expression is spatially repressed as the construct of *ProBRL3* was reduced in size, starting from a loss in the QC (*ProBRL3-1098::GUS*) to a complete loss in the roots (*ProBRL3-498::GUS*). In the shorter constructs, *BRL3* continues to be present in the leaf vascular tissue (*ProBRL3-384::GUS*) and ends in a complete abolishment of *BRL3* expression (*ProBRL3-218::GUS*).

As for *ProBRL3::GUS*, the expression pattern of *ProBRL1::GUS* was examined in 6-day-old seedlings of two representative and independent T_4_ homozygous lines for each construct generated. The longest truncated construct analyzed (*ProBRL1-1641::GUS*) showed GUS expression in the differentiation zone and in the tip of lateral roots, while no GUS expression was detected in the root meristem (Supplementary Fig. S1B, C). Progressively shorter promoter constructs, *ProBRL1-978::GUS* and *ProBRL1-790::GUS*, resulted in a loss of GUS expression in the tip of lateral roots, whereas in the differentiation zone GUS expression was still present (Supplementary Fig. S1F, G, J, K). In the *ProBRL1−479bp::GUS* and *ProBRL1-334::GUS* deletions, that are missing parts of the 5'-untranslated region (UTR), the expression in the root was completely abolished (Supplementary Fig. S1N, O, R, S). These results suggest that the region between base pairs −790 and −479 contains regulatory elements needed for *BRL1* expression.

### Brassinosteroids control the expression of *BRL3* but not *BRL1*

The expression levels of *BRL3* are known to be repressed by BRs (Mussig *et al.*, 2002; Vert *et al.*, 2005; Sun *et al.*, 2010; Yu *et al.*, 2011). Therefore, the effects of BRs on the expression pattern of *BRL3* were investigated. The transgenic lines described above were treated with 4nM BL for 48h. Previous publications already reported that treatments for 24h and 48h, using physiological BL concentrations (below the *K*_d_ of the receptors), showed effects on root expression even when there were no dramatic morphological effects present (González-García *et al.*, 2011). Analysis of BL-treated *ProBRL3-1719::GUS* roots showed a shifted and diffuse expression in the stele and in the two protophloem cell files (Fig. 1E), whereas the expression in the root differentiation zone, in lateral roots, and in the QC (Fig. 1D, E) was similar to that in the untreated plants (Fig. 1B, C). In the shorter promoter deletion lines (*ProBRL3-1098::GUS*, *ProBRL3-755::GUS*, *ProBRL3-498::GUS ProBRL3-384::GUS*, and *ProBRL3-218::GUS*), no significant differences were observed in *BRL3* expression when treated with BL (Fig. 1H, I, L, M, P, Q, T, U, X, Y). However, GUS expression in the two protophloem cell files of BL-treated *ProBRL3-1098::GUS* and *ProBRL3-755::GUS* was significantly down-regulated (Fig. 1I, M). This region between base pairs −1719 and −1098 contains a BRRE at base pair −1441, capable of binding BES1 and BZR1 transcription factors, suggesting a specific regulatory role for BRs in the expression of *BRL3.* Thus, these results indicate that BR modulates the expression of *BRL3* in the root, since *BRL3* expression in other vascular parts remained unchanged (Fig. 2). Conversely, no significant changes in the expression of *ProBRL1::GUS* upon BL treatment were observed in any of the transgenic lines generated (Supplementary Figs S1, S2).

### Cell-specific and dose-dependent control of *BRL3* transcription by BRs

**Fig. 3:**
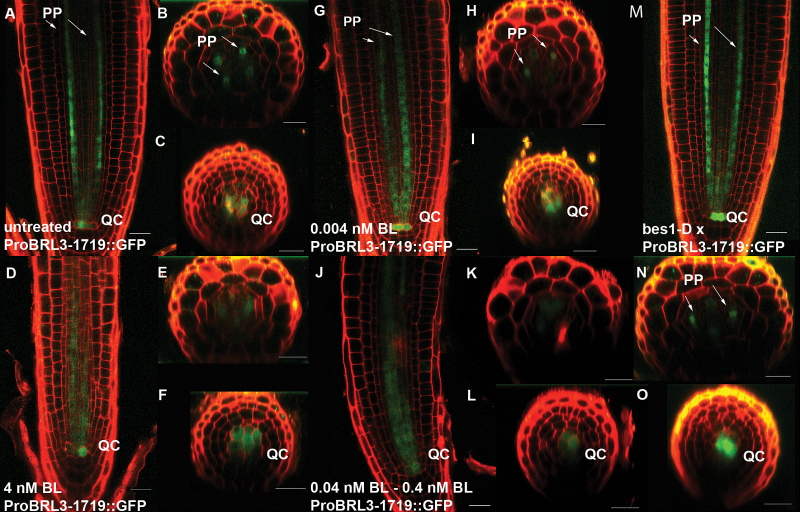
*BRL3* expression pattern in the root meristem is BR dose dependent. (A–F) Confocal images of primary roots expressing *ProBRL3-1719::GFP* in the root differentiation and meristematic zone of 6-day-old P*roBRL3-1719::GFP* transgenics with and without 4nM BL treatment for 48h. PP, protophloem; QC, quiescent center. (A) Untreated lines showed expression in the protophloem cell files at the transition zone where protophloem differentiates, and in the QC. (B, C) Transversal images of the meristem and the QC of untreated lines. (D) Lines treated with 4nM showed expression in the QC and in a diffuse pattern in the stele. (E, F) Transversal images of the meristem in 4nM BL-treated lines. (G–L) *ProBRL3* transgenics treated with increasing concentrations of BL (0.004–4nM continuous treatment) (G) *ProBRL3-1719::GFP* treated with 0.004nM BL showed an increased and expanded expression in the protophloem cell files towards the QC, where the GFP reporter is also expressed. (H, I) Transversal images of the meristem and the QC of *ProBRL3-1719::GUS* after treatment with 0.004nM BL. (J) *ProBRL3-1719::GFP* treated with 0.04–0.4nM BL showed a reduced and misplaced expression in the stele and repression in the QC. (K, L) Transversal images of the meristem and the QC after treatment with 0.04–0.4nM BL. (M–O) *ProBRL3-1719::GFP* transgenics crossed to *bes1-D* lines showed increased expression in the protophloem cell files towards the QC and in the QC, similar to transgenics treated with 0.004nm BL. (N, O) Transversal images of the meristem and the QC of *ProBRL3-1719::GUS* crossed to *bes1-D.* Scale bar=20 µm.

To understand how BRs affect the expression pattern of *BRL3* within the root meristem in more detail, confocal visualization of GFP in the ProBRL3::GFP deletion were counterstained with PI to label the cell walls. Investigating *ProBRL3-1719:: GFP* in the meristem using confocal microscopy revealed specific expression of *BRL3* in the protophloem cell files at the transition zone where undifferentiated protophloem starts to differentiate, as well as in the QC (Fig. 3A–C). Upon 48h of BL treatment of the transgenic line *ProBRL3-1719::GFP,* a shift in expression from the protophloem cell files into the stele and towards the QC was observed (Fig. 3D–F). Due to a spatial shift in expression pattern of *ProBRL3-1719::GFP* in the stele, its specific expression at the transition zone, where protophloem starts to differentiate, could not be detected.

Next a dose-dependent control of *BRL3* expression by BRs in the primary root was investigated by treating *ProBRL3*-1719 reporter lines with different concentrations of BL. At growth-promoting concentrations of BRs (0.004nM) (González-García *et al.*, 2011), the expression of *BRL3* was also enhanced (Fig. 3G–I). The *ProBRL3::GFP* was extended towards the root transition zone and along the protophloem cell files towards the QC (Fig. 3G). A moderate shift of *ProBRL3::GUS* expression into the stele was observed (Fig. 3G–I). In contrast, the root growth-inhibitory BL concentrations (>0.004nM) repressed the *BRL3* expression in the two protophloem cell files while *BRL3* expression was spatially shifted towards the stele and the QC (Fig. 3J–L). The fact that low BL concentrations promote BRL3 in the protophloem cell files, while it is strongly repressed at higher BL concentrations, indicates that *BRL3* regulation by BR follows the same trend as the effect in BRs in root growth. Furthermore, our data reveal that *BRL3* transcription levels in the root apex are tightly regulated by BRs.

### BES1 directly targets and drives the expression of *BRL3* in specific cell types in the root

The presence of a BRRE (base pair −1441) and/or an E-box (base pair −892) in the 5'-flanking region of *BRL3* prompted us to investigate whether BRs might regulate *BRL3* transcription via direct binding of BES1 and/or BZR1 transcription factors. It could be hypothesized that BES1 is able to regulate the specific expression of *BRL3* based on the observation that high levels of BRs repress and/or misexpress *BRL3* in protophloem cells and vascular cells, respectively, whereas low levels promote its expression. The differential role of BES1 over BRZ1 in the control of QC function in the root (Vilarrasa-Blasi *et al.*, 2014) and the fact that the binding of active BES1 to BRREs appeared to be much stronger than to E-boxes (Yin *et al.*, 2005) point to BES1 as the most suitable factor regulating *BRL3* receptors.

To investigate the functional role of BES1 in the BR-regulated *BRL3* expression, *ProBRL3-1719::GFP* was analyzed by genetic crosses with the BR-activated *bes1-D* mutants and an inducible *bes1*-*D* line (*35S::Bes1-D:GR*). In agreement with physiological data (0.004nM of BR treatment for 6 d) in *ProBRL3-1719::GFP* plants, the *ProBRL3-1719::GFP×bes1-D* plants exhibited a similar expression pattern of *ProBRL3::GFP* in the meristem. *BRL3* expression showed an increased and continued expression in the protophloem cell files and in the QC, and a spatial shift to the stele (Fig. 3G–I, M–O). In contrast, the expression in *ProBRL3-1719::GUS* plants in the background of overexpressing *bes1-D*-inducible lines resembled the *ProBRL3::GFP* expression when treated with high BR levels, showing a spatial shift of *BRL3* towards the stele and the QC (Fig. 3D, J; Supplementary Fig. S4). These results support the idea of a dose-dependent BR-regulated *BRL3* expression pattern in the root meristem mainly based on active BES1 protein levels.

### BES1 binds to the BRRE present in the promoter of *BRL3*

Next, the BRRE at base pair −1441 present in the 5'-flanking region of *BRL3* seemed to be an important regulatory element in response to BL, and previous results already demonstrated that *ProBRL3-1719::GFP* expression is modulated by BES1. It might be obvious that BES1 regulates *BRL3* via binding to the BREEs. However, an identified E-box at base pair −892 might also play an additional role in *BRL3* regulation. Additionally, BES1 is known to bind both the BRRE and E-box (Yu *et al.*, 2011). Therefore, we further investigated whether BES1 binds to both the BRRE and E-box.

**Fig. 4:**
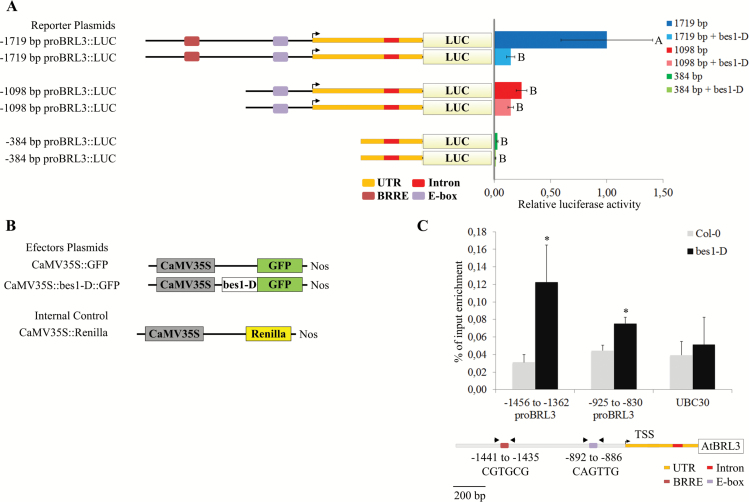
*BES1* regulates the *BRL3* expression pattern in the root meristem through binding to the BRRE. (A, B) BES1 represses *BRL3* expression through binding the BRRE. In a luciferase reporter gene assay, protoplasts of Arabidopsis have been co-transfected with either *ProBRL3-1719::LUC*, *ProBRL3-1098::LUC*, or *ProBRL3-384::LUC* and either 35S::*bes1-D*:GFP or the empty vector 35S:GFP (control), and additionally with 35S::Renilla. The transient transactivation assays were done in biological triplicates and data were normalized for Renilla activity (Grotewold *et al.*, 2000). The ratio was calculated as the ratio of each treatment and the treatment of the longest construct used without the repressor. The plotted diagram shows the arithmetic means and the SEM, demonstrating a strong repression of *BRL3* due to the binding of *BES1* to the BRRE in *ProBRL3-1719::LUC*. Letters indicate significant differences in *ProBRL3*-driven luciferase intensity using a one-way ANOVA followed by a Tukey’s test (*P*<0.05; Tukey’s least significant difference). (C) The 5'-flanking region of *BRL3* containing the BRRE was enriched in the ChIP experiment using antibodies against GFP. BES1 ChIP assays showed strong enrichment at the *BRL3* promoter region containing the BRRE (base pairs −1441 to −1435) and a low enrichment in the region containing an E-box (base pairs −892 to −886). The position and the sequence of the BRRE and E-box elements present in the *BRL3* promoter are shown on the bottom of the scheme. The arrows indicate the primers’ annealing positions. Results are represented as percentage input; the error bars indicate the SD of the data obtained from three technical replicates. As a negative control, *UBC30* has been used. Statistical analysis of differences between fragments of the *ProBRL3* promoter and internal negative controls (*UBC30*) was performed using Student’s *t*-test. Asterisks refer to a significant difference of *P<0.05. Two independent biological replicates gave the same result (Supplementary Fig. S3).

To elucidate whether *BES1* regulates the expression of *BRL3*, a reporter gene assay and ChIP experiments were performed. In the former, Arabidopsis protoplasts were co-transformed with both 35S::*bes1-D*:GFP (effector gene) and different *BRL3* promoter deletion constructs (*ProBRL3-1719*, *ProBRL3-1098*, and *ProBRL3-384*) controlling the luciferase gene (reporter gene). The construct with 35S::GFP was used as a control. Co-transfected protoplasts using *ProBRL3-1719::LUC* and 35S::*bes1-D*:GFP showed a strong reduction in the luciferase activity compared with the co-transfected combination using the control. The *ProBRL3-1098::LUC* or *ProBRL3-384::LUC* constructs did not show significant luciferase activity, neither in co-transfection with overexpressed BES1 nor in co-transfection with the control (Fig. 4A, B).

The ChIP experiment was performed in 35S::*bes1-D*:GFP and wild-type plants using an anti-GFP antibody. The BES1-D ChIP was performed similarly to previous reported work (Vilarrasa-Blasi *et al.*, 2014). In the area of the BRRE (base pair −1441), an enrichment of BES1 was detected, whereas in the region between base pairs −1098 and −755, containing the E-box (base pair −892), only a slight enrichment was observed (Fig. 4C; Supplementary Fig. S3). This indicates that BES1 primarily regulates *BRL3 in vivo*, binding the BRRE at position −1441; although additional regulatory effects exhibited by BES1 bound to the E-box cannot be completely ruled out. In summary, these results address the BR-regulated expression pattern of *BRL3* in the root meristem, which is basically based on BES1 binding to the BRRE at position −1441.

## Discussion

The *BRL3* and *BRL1* genes have been described as *BRI1* homologs (serine/threonine kinase receptors) capable of binding BRs with high affinity and are specifically expressed in the plant vasculature (Caño-Delgado *et al.*, 2004). Histological analysis of a 750bp promoter fragment of *BRL3* fused to a GUS reporter revealed expression in the two protophloem cell files of the Arabidopsis primary root (Caño-Delgado *et al.*, 2004). The recent demonstration of the expression in the vascular tissue and their close homology to the BRI1 receptor led to the proposition that BRL3 as well as BRL1 might also have a functional role in vascular development (Caño-Delgado *et al.*, 2004; Fàbregas *et al.*, 2013). Interestingly, the root length analysis of 6-day-old seedlings showed that *bak1-3* roots are significantly shorter than those of wild-type Col-0 plants (Nam and Li, 2002; Albrecht *et al.*, 2008; Fàbregas *et al.*, 2013), and *brl1 brl3 bak1-3* triple mutants enhanced the *bak1-3* short root phenotype (Fàbregas *et al.*, 2013). In addition, the triple mutant *brl1 brl3 bak1-3* also exhibited a wider stele than Col-0 wild-type and *bak1-3* plants under normal conditions. This points to an involvement of *BRL3* and/or *BRL1* in vascular root development and supports the importance of *BRL3* for BR-mediated root growth.

In addition, recent studies showed that *BRL3* is a BZR1 putative target repressed by BR (Sun *et al.*, 2010) and a down-regulated BR putative target of *BES1* (Yu *et al.*, 2011), whereas *BRL1* is only detected as a non-BR-regulated BZR1 putative target (Sun *et al.*, 2010). Nevertheless, the specific mechanisms of the spatial and temporal regulation of *BRL3* and *BRL1* expression patterns within the root vascular tissue remained unknown. Further identification of regulatory elements, factors driving the expression of *BRL3* and *BRL1,* and crosstalk with other signaling pathways is fundamental to understanding vascular development. In this study, a detailed expression analysis for *BRL3* and *BRL1* and their 5' regulatory regions essential for proper gene expression has been carried out. In addition, this result confirm that the expression of *BRL3* in the root vasculature underlies a dose-dependent hormone-regulatory mechanism.

### Tissue-specific expression of *BRL3* and *BRL1*

This study demonstrates a highly specific expression pattern for *BRL3* and *BRL1* throughout the plant vascular tissue. *BRL3* showed expression in the vascular tissues and the tip of the cotyledons, in the shoot apex, in lateral root primordia, in the differentiation zone, in the two protophloem cell files, and in the QC within the meristematic zone. The *BRL3* promoter domain ranging from base pair −1719 to −1098 as well as the region from base pair −1098 to −755 5' of the translational start codon has been demonstrated to contain essential regulatory elements to drive *BRL3* in different vascular tissues. The minimal promoter length for *BRL3* expression in the root has been confirmed to be 755bp. In transgenic plants carrying promoter constructs shorter than −755bp, the expression in the root was completely abolished, and in deletions generated close to the translational start codon the expression was completely lost in seedlings, indicating the presence of fundamental regions within the 5'-UTR.

### BR-regulated expression of *BRL3* in the root vasculature is dose dependent

The characterization of *ProBRL3-1719::GUS* lines in response to 4nM BL for 48h revealed a shift in the expression pattern in the root meristem. Whereas native *BRL3* was expressed at the transition zone between undifferentiated and differentiated protophloem in the protophloem cell files and the QC, BL treatment expanded the expression from the protophloem cell files to encompass other stele cell types. However, the same treatment in *ProBRL3-1098::GUS* and *ProBRL3-755::GUS* lines did not drive the expression into the stele, but showed a significant reduction of *BRL3* expression in the protophloem cell files. Interestingly, the 5' region between base pairs −1719 and −1098 and between −1098 and −755 contains a BRRE and an E-box, respectively, elements that can be bound by BES1 and/or BZR1 proteins. It is worth mentioning that BRs regulate vascular differentiation, in particular promoting xylem and repressing the formation of phloem (Ibanes *et al.*, 2009).

These results show: (i) that BRs regulate the expression of *BRL3* receptor gene transcripts; and (ii) that this regulation is BR dose dependent. The expression pattern of *BRL3* differs significantly when subjected to different levels of BL; likewise the root meristem needs an equilibrated BR signaling to maintain its length (González-García *et al.*, 2011). For instance, very low levels of BL (0.004nM for 6 d) in *ProBRL3-1719::GUS* increased the expression in the protophloem cell files at the transition zone and an expansion towards the QC. This concentration has recently been demonstrated to promote both root epidermal cell number and size of the Arabidopsis primary root meristem (González-García *et al.*, 2011). In addition, BRs are known to promote cell differentiation (Iwasaki and Shibaoka, 1991; Yamamoto *et al.*, 1997). Thus, it can be assumed that low levels of BRs not only increase the epidermal cell number and size of the meristem but also promote the differentiation of phloem. This observation is opposite to the reported function for BRs in repressing phloem differentiation (Fukuda, 1997). However, recent studies indicate opposing effects of BR signaling in terms of root growth, depending on the tissue on which BR is acting (Vragovic *et al.*, 2015). In the transgenic promoter deletion lines with the GUS/GFP reporter system, *ProBRL3 −1719::GFP*, *−1098::GFP*, and *−755::GFP*, treatment with high levels of BL (≥0.04nM) reduced the marker expression in the protophloem cell files, which could be a consequence of a direct repression caused by BRs and/or a repressed number of differentiated phloem cells. In addition, increasing BR concentrations significantly reduced the root length of wild-type plants (González-García *et al.*, 2011) while *brl1brl3bak1-3* triple mutant plants showed resistance to BR-mediated root shortening (Fàbregas *et al.*, 2013).

Interestingly, and despite the similarity of BRL1 and BRL3 receptors (Caño-Delgado *et al.*, 2004; Fàbregas *et al.*, 2013), these results indicate that *BRL1* transcription appeared not to be regulated by BRs. Moreover, an additional study already reported that *BRL1* was detected as a non-BR regulated *BZR1* putative target (Sun *et al.*, 2010). Further biochemical studies involving the dephosphorylated and phosphorylated forms of *BES1* will be necessary to understand the different regulatory mechanisms for these two functionally homologous vascular receptors during plant growth and development.

### BRs regulate *BRL3* expression through binding of BES1 to a BRRE

These findings reveal a role for BES1 as an important factor regulating the expression pattern of *BRL3* in the root meristem. Interestingly, checking the expression pattern of *BRL3* and *BES1* in the eFP browser (http://bar.utoronto.ca/efp/cgi-bin/efpWeb.cgi) showed a correlation between low transcriptional levels of *BES1* and high levels of *BRL3,* and between very high levels of *BES1* and low levels of *BRL3* (Brady *et al.*, 2007) (Supplementsry Fig. S5).

Recently, Chaiwanon and Wang (2015) reported that the BZR1–yellow fluorescent protein (YFP) fusion protein expressed from either the BZR1 promoter or the constitutive 35S promoter accumulated at a low level in the nuclei of the stem cell region but at a higher level in the nuclei of epidermal cells in the transition and elongation zone as well as in the phloem. In addition, BES1–GFP under the endogenous promoter showed a similar pattern to BZR1–YFP (Chaiwanon and Wang, 2015). This correlates with the role of BRs in vascular differentiation and also supports the hypothesis of this study that in the case of *BRL3*, BES1 seems to act as an activator of *BRL3* at low levels whereas at elevated levels, BES1 acts as a repressor of *BRL3.* Moreover, Jiang *et al.* (2015) recently identified a novel long isoform of BES1, called BES1-L. The BES1-L–GFP line presented in their study showed an expression pattern in the root meristem similar to that of the *ProBRL3-1719::GUS* line analyzed in this study.

*In vivo* experiments, such as ChIP and a luciferase reporter gene assay, confirmed the direct interaction of BES1 and the BRRE present in *BRL3*. ChIP assays showed that BES1 binds to the *BRL3* promoter in a region comprising a BRRE *cis*-element. In the region containing an E-box element, only a slight enrichment of *BES1* has been observed, indicating that the E-box plays a minor, perhaps additive, role in *BRL3* regulation. In addition, it has already been reported that the binding affinity of BES1 for a BRRE is stronger than for E-boxes (Yin *et al.*, 2005).

In the luciferase reporter gene assay, BES1 showed a significant reduction in the expression of the luciferase reporter in protoplasts co-transfected with *ProBRL3-1719::LUC* and 35S:*:bes1-D*:GFP when compared with co-transfections of *ProBRL3-1719::LUC* and 35S::GFP alone. In summary, this study reveals that *BRL3* expression in the root meristem is BES1 dose dependent and mediated mainly by the binding of BES1 to the BRRE present in the promoter of *BRL3*.

### A role for BES1 in spatiotemporal control of BRL3 receptors in the stele

This study reveals that the levels of active BR-regulated BES1 control the spatiotemporal transcription of the BRL3 receptors in the root meristem. While the BES1 and BZR1 transcription factors can bind to the BRRE and the E-box, resulting in either activation or repression of the expression of their target genes (Sun *et al.*, 2010; Yu *et al.*, 2011), the mechanisms by which BES1/BZR1 mediate the BR-repressed gene expression are not well understood. It is well known that BES1 and BZR1 inhibit many genes involved in BR biosynthesis and signaling, probably as a feedback inhibition mechanism. Activation of BR signaling inhibits BR biosynthesis and perception through direct repression of *DWF4*, *CPD*, *BRI1*, and other genes by BES1 and BZR1 (Mathur *et al.*, 1998; Noguchi *et al.*, 1999; Choe *et al.*, 2002; Mora-García *et al.*, 2004; Sun *et al.*, 2010; Clouse, 2011; Ye *et al.*, 2011; Yu *et al.*, 2011). BR-mediated gene regulation requires BES1/BZR1 interaction with other partners such as transcription factors, histone-modifying enzymes, and transcription elongation factors (Yin *et al.*, 2005; Yu *et al.*, 2008; Li *et al.*, 2009, 2010; Zhang *et al.*, 2014). Based on these results, a feedback mechanism for *BRL3* regulation via BR signaling mediated by BES1 protein can be proposed.

The key factor in *BRL3* regulation is the level of BES1 present in active BR signaling. When the level of the nuclear-localized BR-activated transcription factor BES1 is low, binding to the BRRE box in the *BRL3* promoter results in an increase of *BRL3* expression in the root meristem. However, at high BES1 levels, binding of the BRRE box suppresses the expression in the two protophloem cell files. Thus the spatiotemporal *BRL3* expression is dependent on BES1 levels.

In addition, it has recently been proposed that the dose-dependent opposite effects of exogenous BR are due to a requirement for different BR levels in different developmental zones (Chaiwanon and Wang, 2015). Interestingly, BZR1 is activated by endogenous BR in a graded pattern along developmental zones and BRs act antagonistically with auxin on BZR1 nuclear localization, transcriptomic response, and cell elongation in a developmental zone-specific manner (Chaiwanon and Wang, 2015). Collectively, it can be argued that the repression of *BRL3* in the two protophloem cell files functions in co-ordinating BR-mediated root development, especially during the differentiation of phloem cell files. Thus the integration of cell type-specific signaling events in response to environmental stimuli is important to understand plant growth and development completely.

From the biotechnological point of view, identification of the *cis*-acting regulatory elements is gaining great importance because of the emergence of tools for genome editing such as the zinc-finger nucleases (ZFNs), the transcriptional activator-like effector nucleases (TALLENs), and the clustered regularly interspaced short palindromic repeats/Cas9 (CRISPR/CAS) system. Any modification of the promoter architecture of BR receptors via insertions or deletions in the E-box and/or BRRE can enable the modification of signaling events within the BR pathway. It is very well known that BRs play an important role in plant development including plant architecture, vascular differentiation, and flowering, and in the physiological responses such as tolerance to biotic and abiotic stress. In addition, the present work reporting the identification of promoter regions important for vascular expression may open the door to the identification and validation of new *cis*-regulatory elements important for plant vascular development.

## Supplementary data

Supplementary data are available at *JXB* online

Figure S1. Promoter deletion analysis of *BRL1* in the root.

Figure S2. Promoter deletion analysis of *BRL1*.

Figure S3. BES1 was enriched in the 5'-flanking region of *BRL3* containing the BRRE.

Figure S4. *BRL3* expression pattern in the root meristem is BES1 dose dependent.

Figure S5. Relative and comparative expression patterns of expression for *BRL3* and *BES1.*

Table S1. Primer sequences

Supplementary Data
