# Supplementary material for: BES1 regulates the localization of the brassinosteroid receptor BRL3 within the provascular tissue of the Arabidopsis primary root
Source: J Exp Bot. 2016 Aug 10;67(17):4951–61. doi: 10.1093/jxb/erw258 (PMC5014150; doi:10.1093/jxb/erw258)
Supplement: Supplementary Data [file supp_67_17_4951__index.html]

BES1 regulates the localization of the brassinosteroid receptor BRL3 within the provascular tissue of the Arabidopsis primary root — BES1 regulates the localization of the brassinosteroid receptor BRL3 within the provascular tissue of the Arabidopsis primary root — Supplementary Data 

# BES1 regulates the localization of the brassinosteroid receptor BRL3 within the provascular tissue of the Arabidopsis primary root

## Supplementary Data

Data files

- Supplementary\_figure\_table\_legends.pdf - Supplementary Data
- Supplementary\_figure\_S1.tif - Supplementary Data
- Supplementary\_figure\_S2.tif - Supplementary Data
- Supplementary\_figure\_S3.tif - Supplementary Data
- Supplementary\_figure\_S4.tif - Supplementary Data
- Supplementary\_figure\_S5.pptx - Supplementary Data
- Supplementary\_table\_S1.docx - Supplementary Data
