# Supplementary material for: BES1 regulates the localization of the brassinosteroid receptor BRL3 within the provascular tissue of the Arabidopsis primary root
Source: J Exp Bot. 2016 Aug 10;67(17):4951–61. doi: 10.1093/jxb/erw258 (PMC5014150; doi:10.1093/jxb/erw258)
Supplement: Supplementary Data [file supp_erw258_Supplementary_figure_table_legends.pdf]

BES1 regulates the localisation of the Brasssinostertoid receptor BRL3 within the provascular tissue of the Arabidopsis primary root. *Jorge E. Salazar-Henao, Reinhard Lehner, Isabel Betegón-Putze, Josep Vilarrasa-Blasi, and Ana I. Caño-Delgado*

SUPPLEMENTARY DATA

Fig.S1 Promoter deletion analysis of *ProBRL1* in the Arabidopsis primary root. (A) Schematic diagram of the 5' flanking regions of *ProBRL1*. The figure represents part of the 5' flanking region of *ProBRL3* including the 5' UTR and an intron, labelled in orange and red, respectively. The lower part represents the deletion constructs generated fused to the reporter genes GFP and GUS. (B-U) Histochemical GUS assay in cotyledons, SAM, root differentiation and meristematic zone of 6-day-old *ProBRL1* transgenics with and without 4 nM BL treatment for 48 hrs. Scale Bar: 125µm (B-C) *ProBRL1-1641::GUS* showed expression in the tip of lateral roots and in the differentiation zone. (F-G) *ProBRL1-978::GUS* and (J-K) *ProBRL1-790::GUS* showed expression in the differentiation zone. (N-O) *ProBRL1-479::GUS* and (R-S) *ProBRL1-334::GUS* did not show any expression in the tissues analysed. After treatment with BL an alteration in the expression pattern of *BRL1* in the root was not observed. (D-E) *ProBRL1-1641::GUS* showed expression in the tip of lateral roots and in the differentiation zone. (H-I) *ProBRL1-978::GUS* and (L-M) *ProBRL1-790::GUS* showed expression in the differentiation zone. (P-Q) *ProBRL1-479::GUS* and (T-U) *ProBRL1-334::GUS* did not show any expression in the tissues analysed.

Fig.S2 Promoter deletion analysis of *BRL1*. (A-T) Histochemical GUS assay in cotyledons, SAM, root differentiation and meristematic zone of 6-day-old *ProBRL1* transgenics with and without 4 nM BL treatment for 48 hrs. Scale Bar: 125µm (A-B) *ProBRL1-1641::GUS* showed expression in the veins and the tip of the cotyledons and in the SAM. (E-F) *ProBRL1-978::GUS* and (I-J) *ProBRL1-790::GUS* showed expression in the veins and the tip of the cotyledons. (M-N) *ProBRL1-479::GUS* and (Q-R) *ProBRL1-334::GUS* did not show any expression in the tissues analysed. After treatment with BL an alteration in the expression pattern of *BRL1* in the cotyledon and in the SAM was not observed. (C-D) *ProBRL1-1641::GUS* +BL showed expression in the veins and the tip of the cotyledons and in the SAM. (G-H) *ProBRL1-978::GUS* and (K-L) *ProBRL1-790::GUS*

showed expression in the veins and the tip of the cotyledons. (O-P) *ProBRL1-479::GUS* and (S-T) *ProBRL1-334::GUS* did not show any expression in the tissues analysed.

Fig.S3 BES1 was enriched in the 5' flanking region of *BRL3* containing the BRRE. ChIP assays showed a strong enrichment in the *BRL3* promoter region containing the BRRE (-1441 to -1435 bp) and a low enrichment in the region containing an E-Box (-892 to -886). Resulting data of a chromatin immunoprecipitation (ChIP) experiment showed enrichment of BES1 in the promoter region of *BRL3* containing the BRRE (-1441 to -1435 bp). In addition between the region -892 and -886 bp, containing an E-box, a low enrichment of BES1 was detectable. The position and sequence of the BRRE and E-box elements present in the *BRL3* promoter are shown on the bottom of the scheme. The arrows indicate the primers' annealing positions. Results are represented as % Input, the error bars indicate the standard deviation of the data obtained from three technical replicates. As an internal negative control the UBC30 have been used. Statistical analysis of differences between fragments of *ProBRL3* promoter and negative control (Col-0) was performed using Student's t-test. Asterisks refer to a significant difference of $*p < 0.05$. Two independent biological replicates have the same result.

Fig.S4 *BRL3* expression pattern in the root meristem is BES1 dose dependent. Histochemical GUS assay followed by mPS-PI staining and imaging using a confocal microscope in the root differentiation and meristematic zone of 6-day-old *ProBRL3-1719::GUS* and *ProBRL3-1719::GUS* crossed to 35S:*bes1-D:GR.* (A) *ProBRL3-1719::GUS* showed expression in the protophloem cell files at the transition zone where protophloem differentiate, and in the QC. (B) *ProBRL3-1719::GUS* crossed to 35S::*bes1-D*:GR lines showed expression in the QC and a diffused expression pattern in the stele, similar to transgenics treated with 4 nM BL for 48 hrs.

Fig. S5 Relative and comparative expression patterns of expression for BRL3 and BES1. (A-B) Expression of *BRL3* and BES1 in relative expression mode, showing co-localization between both expression patterns. (C) Expression of the BES1 versus *BRL3* as visualized in the compare mode, showing that stronger expression (red) of BES1 is associated with low expression of *BRL3* and medium expression (yellow) of BES1 is associated with high expression of *BRL3*.

Table S1. Primer sequences.
