# Supplementary figures and images for: BES1 regulates the localization of the brassinosteroid receptor BRL3 within the provascular tissue of the Arabidopsis primary root
Source: J Exp Bot. 2016 Aug 10;67(17):4951–61. doi: 10.1093/jxb/erw258 (PMC5014150; doi:10.1093/jxb/erw258)

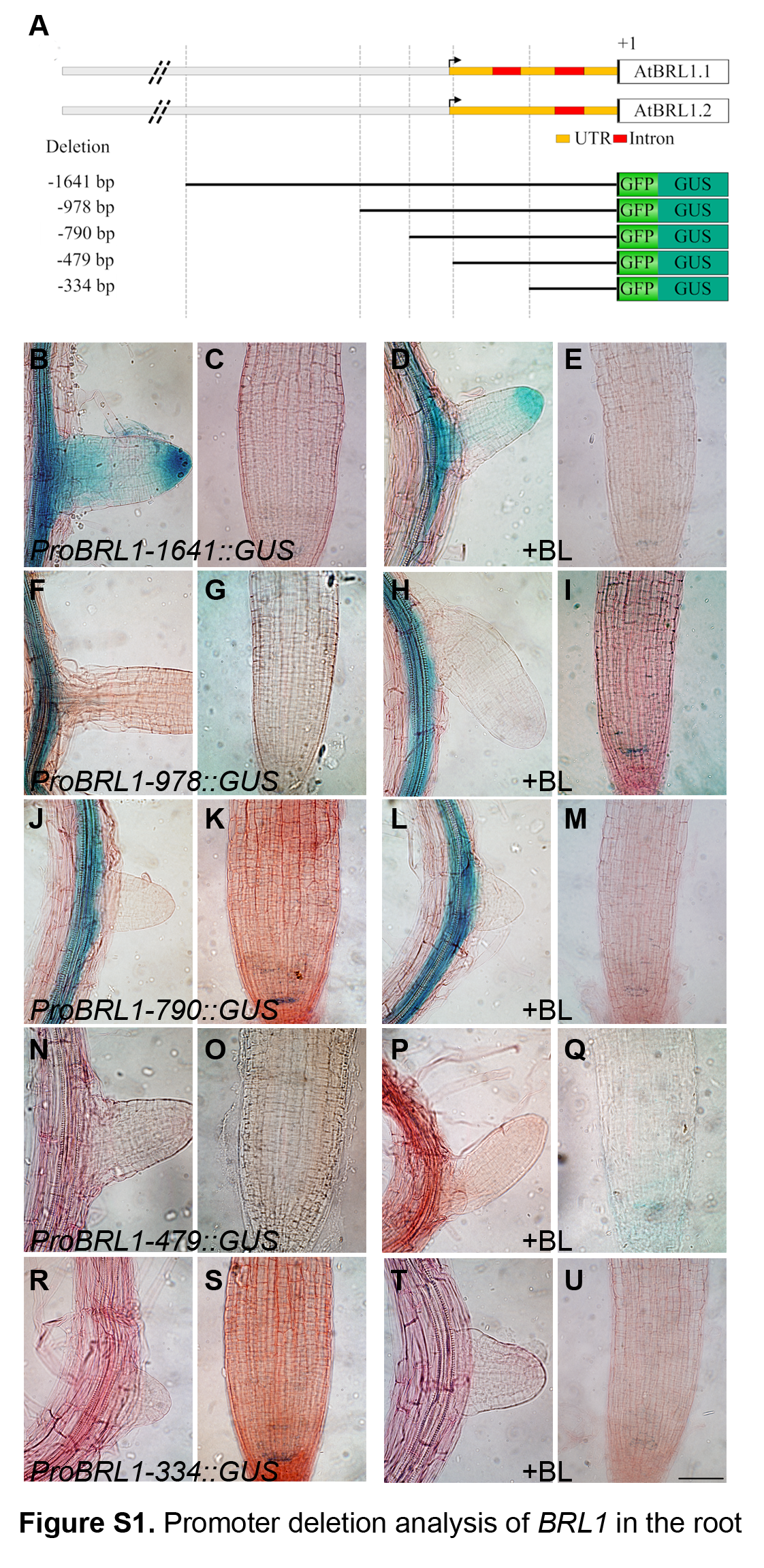

Supplement: Supplementary Data [file supp_erw258_Supplementary_figure_S1.tif]

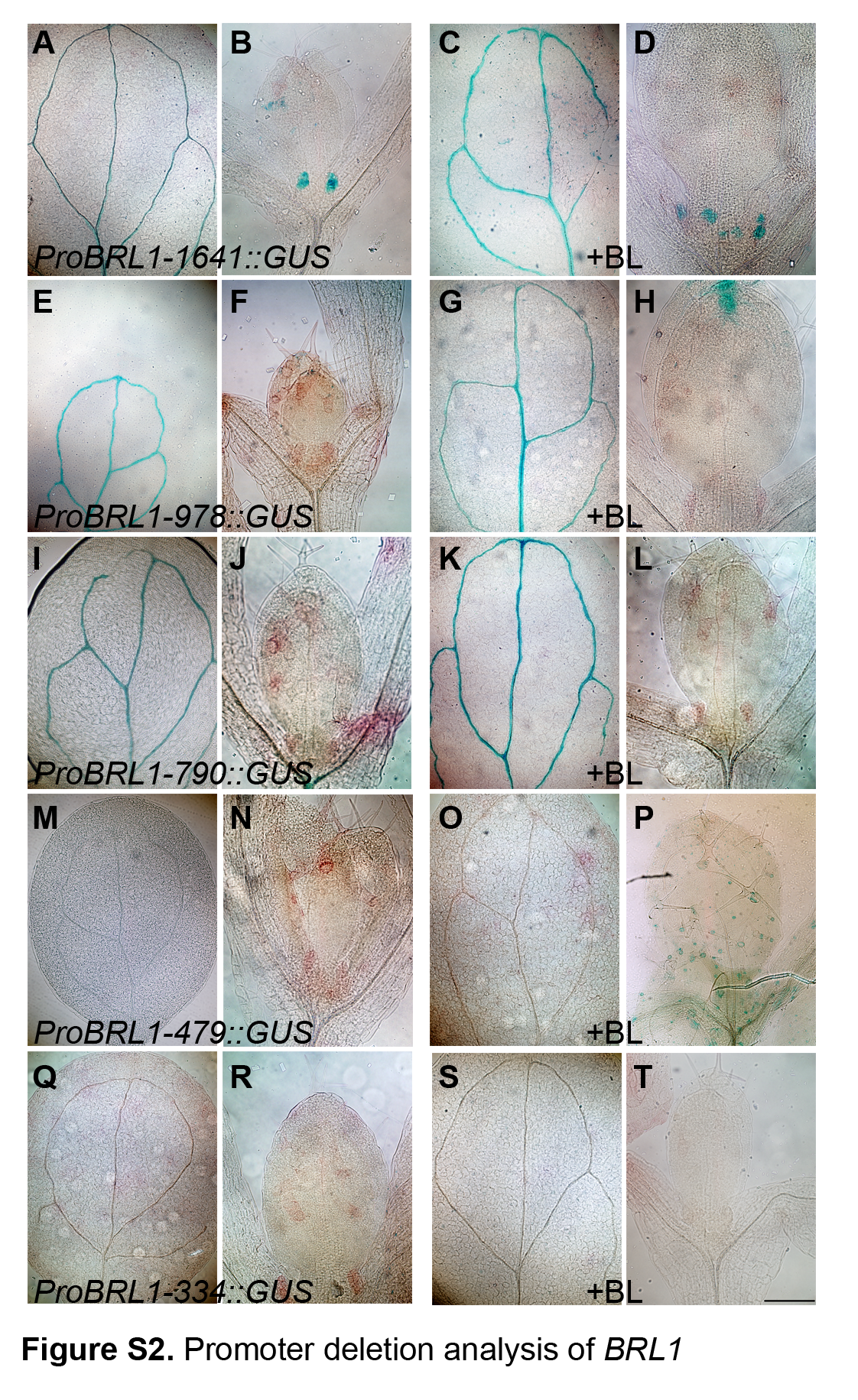

Supplement: Supplementary Data [file supp_erw258_Supplementary_figure_S2.tif]

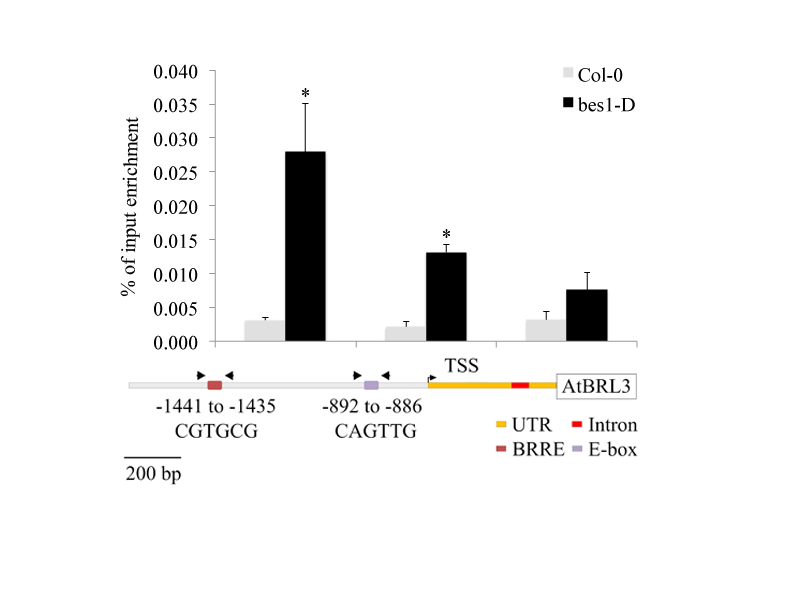

Supplement: Supplementary Data [file supp_erw258_Supplementary_figure_S3.tif]

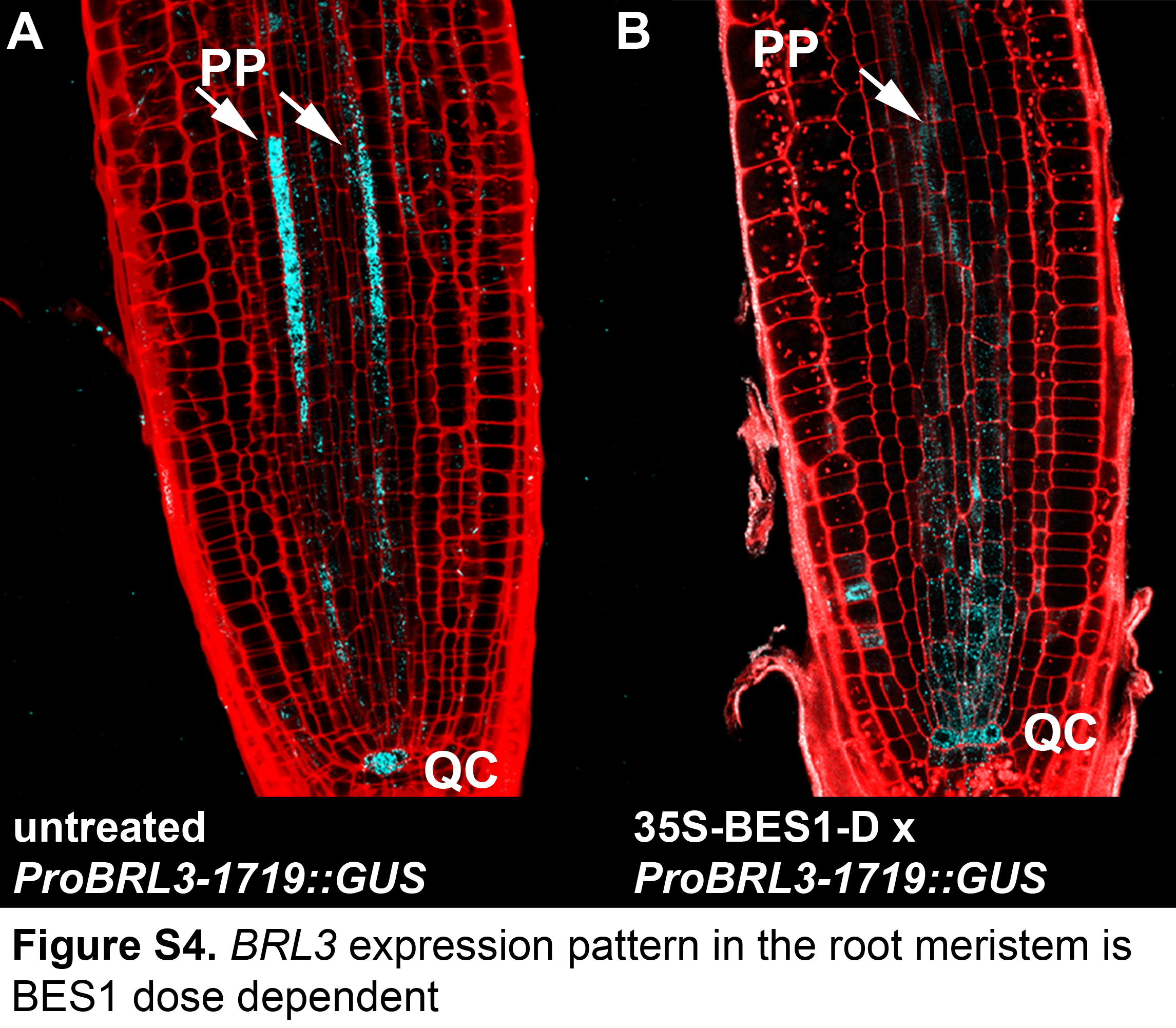

Supplement: Supplementary Data [file supp_erw258_Supplementary_figure_S4.tif]
