# Supplementary material for: BES1 regulates the localization of the brassinosteroid receptor BRL3 within the provascular tissue of the Arabidopsis primary root
Source: J Exp Bot. 2016 Aug 10;67(17):4951–61. doi: 10.1093/jxb/erw258 (PMC5014150; doi:10.1093/jxb/erw258)
Supplement: Supplementary Data [file supp_erw258_Supplementary_figure_S5.pptx]

## Slide 1
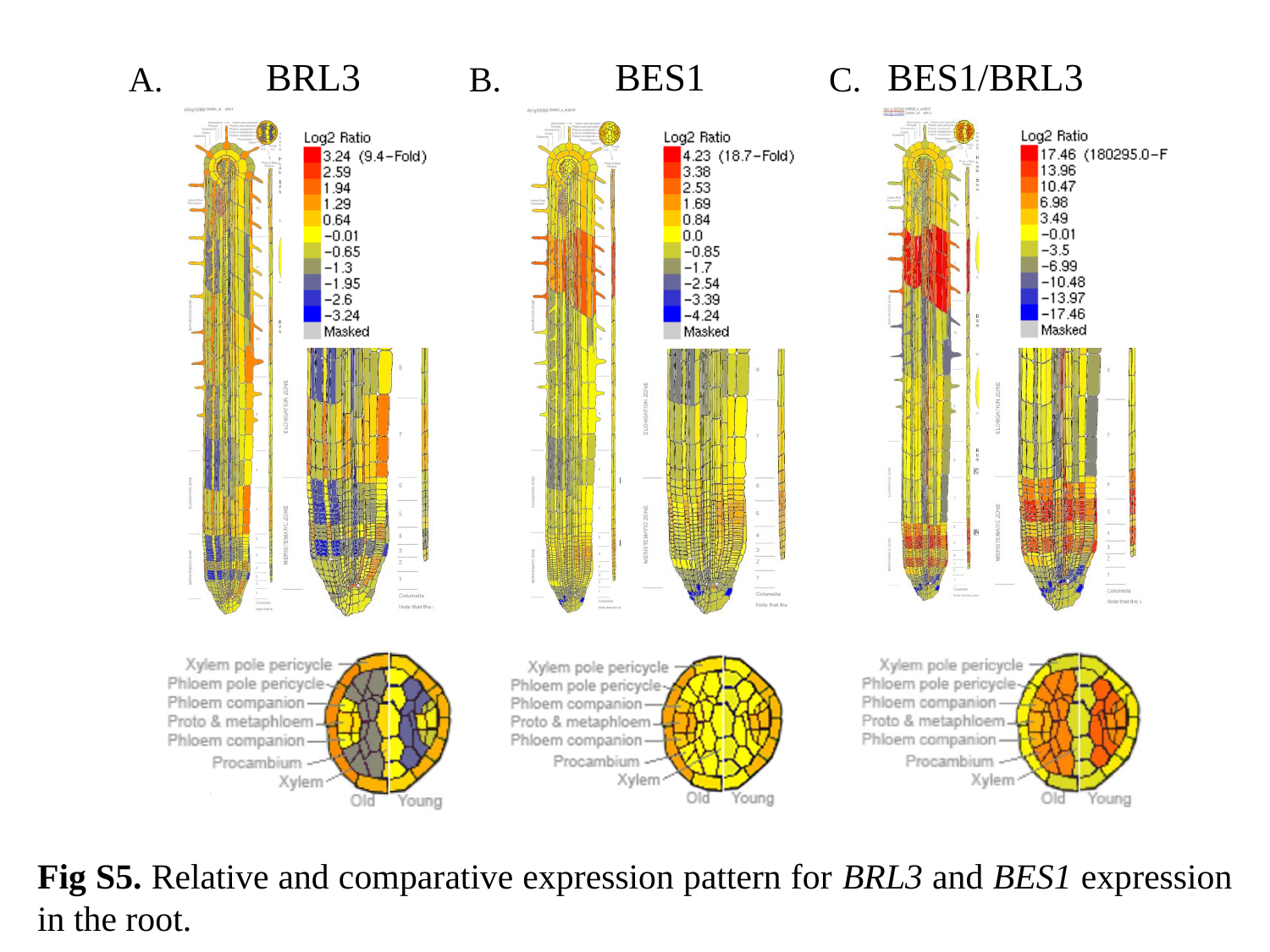

BRL3
BES1
BES1/BRL3
A.
B.
C.
Fig S5. Relative and comparative expression pattern for BRL3 and BES1 expression in the root.
