# Supplementary material for: BES1 regulates the localization of the brassinosteroid receptor BRL3 within the provascular tissue of the Arabidopsis primary root
Source: J Exp Bot. 2016 Aug 10;67(17):4951–61. doi: 10.1093/jxb/erw258 (PMC5014150; doi:10.1093/jxb/erw258)
Supplement: Supplementary Data [file supp_erw258_Supplementary_table_S1.docx]

**Table S1. Primer sequences.**

| **Primer** | **Primer sequence (5’ to 3’)** | **Notes** |
| --- | --- | --- |
| ProBRL1-1641-F | ggggacaagtttgtacaaaaaagcaggctaaaatgctactttggc | Cloning |
| ProBRL1-978-F | ggggacaagtttgtac**aaaaaagcaggct**tcatcaacccaagtaac | Cloning |
| ProBRL1-790-F | ggggacaagtttgtac**aaaaaagcaggct**tgtgacctcacttgcc | Cloning |
| ProBRL1-625-F | ggggacaagtttgtac**aaaaaagcaggct**actcctcaattacgcaa | Cloning |
| ProBRL1-384-F | ggggacaagtttgtacaaaaaagcaggctagacaacctctgttgtc | Cloning |
| ProBRL1-R | ggggaccactttgtac**aagaaagctgggtcatttggcacagcaagag** | Cloning |
| ProBRL3-1719-F | ggggacaagtttgtacaaaaaagcaggctcgtggggattagttgctga | Cloning |
| ProBRL3-1098-F | ggggacaagtttgtacaaaaaagcaggctggaaaccgaactat | Cloning |
| ProBRL3-755-F | ggggacaagtttgtacaaaaaagcaggctgtctcatagttttgtc | Cloning |
| ProBRL3-498-F | ggggacaagtttgtacaaaaaagcaggctacacgcttcctttat | Cloning |
| ProBRL3-384-F´ | ggggacaagtttgtacaaaaaagcaggctgagagacaacactgtcg | Cloning |
| ProBRL3-218-F | ggggacaagtttgtacaaaaaagcaggctcttgaacaagacttg | Cloning |
| ProBRL3-R | ggggaccactttgtacaagaaagctgggtcgttattagcccacaa | Cloning |
| BES1D-F | ggggacaagtttgtacaaaaaagcaggcttaatgaaaagattcttctataattcc | Cloning |
| BES1D-R | gggggaccactttgtacaagaaagctgggtaactatgagctttaccatttcc | Cloning |
| ProBRL3_RG1-F | gaagcgtgagacgttcgtg | ChIP-qPCR |
| ProBRL3_RG1-R | aacgtcccaaagcaaatcat | ChIP-Qpcr |
| ProBRL3_RG2-F | tctcccctaacctgtaaaactca | ChIP-qPCR |
| ProBRL3_RG2-R | cccacctaatagtattcgtgcaa | ChIP-qPCR |
| UBC30-F | caaatccaaaaccctagaaaccgaa | ChIP-qPCR |
| UBC30-R | aacgacgaagatcaagaactgggaa | ChIP-qPCR |
